# The complete plastome sequence of *Diospyros blancoi* A. DC. (Ebenaceae)

**DOI:** 10.1080/23802359.2016.1219647

**Published:** 2016-09-01

**Authors:** Sangjin Jo, Hoe-Won Kim, Young-Kee Kim, Se-Hwan Cheon, Ki-Joong Kim

**Affiliations:** School of Life Sciences, Korea University, Seoul, Korea

**Keywords:** Plastome, tropical fruit, Ebenaceae, *Diospyros blancoi*

## Abstract

The plastome sequences of *Diospyros blancoi* A. DC. (Ebenaceae) were completed in this study (NCBI acc. no. KX426216). The gene order and structure of the *D. blancoi* plastome are collinear with the typical plastome of land plants. The complete plastome size is 157,745 bp in length and consists of a large single-copy region of 87,246 bp and a small single-copy region of 18,323 bp, which are separated by a pair of 26,088 bp-long inverted repeat regions. The overall A-T content of the plastome sequence is 62.6%. The plastome contains 113 genes, of which 79 are protein-coding genes, 30 are tRNA genes, and 4 are rRNA genes. Sixteen genes contain one intron and two genes have two introns. A total of 45 simple sequence loci were identified from the genome. Phylogenetic analysis revealed that *D. blancoi* is a sister group of Primulaceae with 100% bootstrap support.

*Diospyros blancoi* A. DC. is commonly referred to as velvet apple. It is a widely cultivated tropical Asian fruit that originated in Philippine (Kim 2011). But, this is a rare plant in wild population. The fruit and stem of this species is used for both food and timber. *Diospyros blancoi* belongs to the family Ebenaceae of Ericales (Byng et al. 2016). Ericales consist of twenty-two families (Byng et al. 2016). Ericaceae consist of four genera and approximately 800 species (Christenhusz & Byng 2016). Most of them belong to the genus *Diospyros*. The recent studies reported a few plastome from *Diospyrus*, but no sequence data was released from the NCBI so far (Turner et al. 2016; Fu et al. 2016). Furthermore, the two studies did not include the *D. blancoi*. The complete plastome sequence of *D. blancoi* will aid us in developing molecular markers for the identification and improvement of cultivars of this economically important tropical fruit species.

The leaves of *D. blancoi* used in this study were collected from the Korea University greenhouse, where we grew the plants from the seeds that were originally collected from the Philippines. The plants flowered and fruited in the greenhouse. A voucher specimen was deposited in the Korea University Herbarium (KUS acc. no. 2014-0240). Fresh leaves were ground into powder in liquid nitrogen and total DNAs were extracted using the CTAB method (Doyle & Doyle 1987). The DNAs were further purified by ultracentrifugation and dialysis (Palmer 1986). The genomic DNAs are deposited in the Plant DNA Bank in Korea (PDBK acc. no. 2014-0240). The complete plastome sequence was generated using an Illumina HiSeq 2000 system (Illumina, Inc., San Diego, CA). Annotations were performed using the National Center for Biotechnology Information (NCBI) BLAST, DOGMA (Wyman et al. 2004), and tRNAscan-SE programs (Lowe & Eddy 1997). For the phylogenetic analysis, we selected and downloaded 24 complete plastome sequences based on the APG IV system (Byng et al. 2016) from the NCBI database. All the 25 plastome sequences were from super-asterid plants.

The gene order and structure of the *D. blancoi* plastome are similar to those of a typical angiosperm (Shinozaki et al. 1986; Kim & Lee, 2004; Yi & Kim 2012). The complete plastome is 157,745 bp in length, and consists of a large single-copy (LSC) region of 87,246 bp and a small single-copy (SSC) region of 18,323 bp separated by two inverted repeats (IR) of 26,088 bp. The published *D.* plastome sizes are ranged from 157,300 bp to 157,784 bp (Turner et al. 2016; Fu et al. 2016). Therefore, the plastome size of *D. blancoi* also belongs to the suggested range. The plastome of *D. blancoi* comprises 113 unique genes (79 protein-coding genes, 30 tRNA genes, and 4 rRNA genes). Seven protein-coding, seven tRNA, and four rRNA genes are duplicated in the IR regions. The major portion of the *D. blancoi* plastome consists of protein-coding genes (54.8%), tRNA genes (1.8%), and rRNA genes (5.8%). The average A-T content of the plastome is 62.6%, whereas that in the LSC, SSC, and IR regions is 64.7%, 69.1%, and 56.9%, respectively. Sixteen genes have one intron and two genes, *ycf3* and *clpP*, comprising two introns. A total of 45 simple sequence repeat (SSR) loci, which can be defined as having more than 10 duplications of simple nucleotide(s), are scattered among the plastome. Among these, 40, 3, and 2 are mono-SSR, di-SSR, and tri-SSR loci, respectively. Some of these loci will be useful in identifying cultivars of *D. blancoi*.

To validate the phylogenetic relationships of *D. blancoi* in asterids, we constructed a maximum-likelihood (ML) tree by using 25 super-asterid taxa. Phylogenetic analysis was performed on a data set that included 79 protein-coding genes and 4 rRNA genes from the 25 taxa using RAxML v. 7.7.1 (Stamatakis et al. 2008). The 80 gene sequences (81,895 bp in length) were aligned with the MUSCLE program using Geneious v. 6.1.8 (Biomatters Ltd.). As a result, *D. blancoi* form a clade with Primulaceae with a 100% bootstrap value (Figure 1). This results are similar to the recent updated phylogeny of the group (www.mobot.org/MOBOT/research/APweb/). However, the results are somewhat different form other data (Fu et al. 2016). Fu et al. (2016) data suggested that Primulaceae is the basal family of the Ericales, and Ebenaceae is the sister group to other members of Ebenales. Our complete plastome sequence of *D. blancoi* will be helpful to resolve not only the interfamiliar relationships of Ericales, but also the species relationship within a *Diospyros* (Ebenaceae).

**Figure 1.**
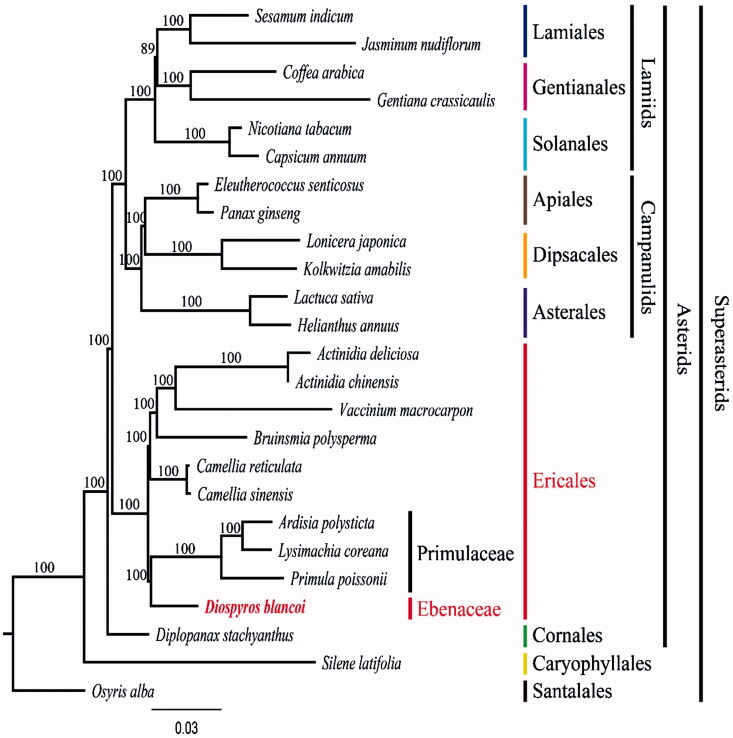
Maximum-likelihood (ML) tree based on 79 protein-coding and 4r rRNA genes from 25 plstomes as determined by RAxML(−ln *L =* −413544.670182). The numbers at each node indicate the ML bootstrap values. Genbank accession numbers of taxa are shown below, *Actinidia chinensis* (NC_026690), *A. deliciosa* (NC_026691), *Ardisia polysticta* (NC_021121), *Bruinsmia polysperma* (NC_030180), *Camellia reticulata* (NC_024663), *C. sinensis* (NC_020019), *Capsicum annuum* (NC_018552), *Coffea arabica* (NC_008535), *D. blancoi* (NC_022408), *Diplopanax stachyanthus* (NC_029750), *Eleutherococcus senticosus* (NC_016430), *Gentiana crassicaulis* (NC_027442), *Helianthus annuus* (NC_007977), *Jasminum nudiflorum* (NC_008407), *Kolkwitzia amabilis* (NC_029874), *Lactuca sativa* (NC_007578), *Lonicera japonica* (NC_026839), *Lysimachia coreana* (NC_026197), *Nicotiana tabacum* (NC_001879), *Osyris alba* (NC_027960), *Panax ginseng* (NC_006290), *Primula poissonii* (NC_024543), *Sesamum indicum* (NC_016433), *Silene latifolia* (NC_016730), and *Vaccinium macrocarpon* (NC_019616).
